# Computational Saturation Mutagenesis Reveals Pathogenic and Structural Impacts of Missense Mutations in Adducin Proteins

**DOI:** 10.3390/genes16080916

**Published:** 2025-07-30

**Authors:** Lennon Meléndez-Aranda, Jazmin Moreno Pereyda, Marina M. J. Romero-Prado

**Affiliations:** 1 Departamento de Bioinformática, Centro de Investigación y Desarrollo Científico del Occidente de México, Centro de Investigación y Desarrollo Cientìfico del Occidente de Mexico (CIDCOM), Guadalajara C. P. 44270, Mexico; lennon.melendez.aranda@gmail.com; 2 Hospital INTER-HOSP, S. A. DE C. V. calle Tuxpan No. 25, Col. Roma Sur, Ciudad de México C. P. 06760, Mexico; jazminmorenopereydal@gmail.com; 3 Departamento de Fisiología, Centro Universitario de Ciencias de la Salud (CUCS), Universidad de Guadalajara, CUCS, Guadalajara C. P. 44340, Mexico

**Keywords:** in silico saturation mutagenesis, calmodulin binding site, phosphorylation site, cytoskeletal regulation

## Abstract

**Background and objectives**: Adducins are cytoskeletal proteins essential for membrane stability, actin–spectrin network organization, and cell signaling. Mutations in the genes *ADD1*, *ADD2*, and *ADD3* have been linked to hypertension, neurodevelopmental disorders, and cancer. However, no comprehensive in silico saturation mutagenesis study has systematically evaluated the pathogenic potential and structural consequences of all possible missense mutations in adducins. This study aimed to identify high-risk variants and their potential impact on protein stability and function. **Methods**: We performed computational saturation mutagenesis for all possible single amino acid substitutions across the adducin proteins family. Pathogenicity predictions were conducted using four independent tools: AlphaMissense, Rhapsody, PolyPhen-2, and PMut. Predictions were validated against UniProt-annotated pathogenic variants. Predictive performance was assessed using Cohen’s Kappa, sensitivity, and precision. Mutations with a prediction probability ≥ 0.8 were further analyzed for structural stability using mCSM, DynaMut2, MutPred2, and Missense3D, with particular focus on functionally relevant domains such as phosphorylation and calmodulin-binding sites. **Results**: PMut identified the highest number of pathogenic mutations, while PolyPhen-2 yielded more conservative predictions. Several high-risk mutations clustered in known regulatory and binding regions. Substitutions involving glycine were consistently among the most destabilizing due to increased backbone flexibility. Validated variants showed strong agreement across multiple tools, supporting the robustness of the analysis. **Conclusions**: This study highlights the utility of multi-tool bioinformatic strategies for comprehensive mutation profiling. The results provide a prioritized list of high-impact adducin variants for future experimental validation and offer insights into potential therapeutic targets for disorders involving *ADD1*, *ADD2*, and *ADD3* mutations.

## 1. Introduction

The *ADD1, ADD2*, and *ADD3* genes encode for ADDA, ADDB, and ADDG proteins (the α-, β-, and γ-adducins), which are essential cytoskeletal regulators involved in maintaining cellular architecture and function. *ADD1* and *ADD3* genes are ubiquitously expressed across a wide range of mammalian tissues. In contrast, *ADD2* gene expression is largely confined to the brain and the hematopoietic system, especially erythrocytes. Two ADDA subunits conform a tetramer with two of ADDB playing a central role in organizing the spectrin–actin cytoskeletal network in erythocytes, podocytes, and others, while ADDG is particularly enriched in epithelial and neuronal cells, contributing to cell–cell junction stability [1,2,3]. Despite their distinct genetic loci, adducins exhibit high sequence homology and typically function as heterodimers or heterotetramers to modulate cytoskeletal dynamics [4]. They cap and bundle actin filaments, stabilizing the spectrin–actin network, which is critical for cell migration, adhesion, and division. Adducin activity is modulated by phosphorylation via PKC and PKA, as well as by calcium-dependent calmodulin binding. Also, phosphorylation mediated by Rho/ROCK decreases adducin’s affinity for spectrin–actin filaments, promoting cytoskeletal disassembly or remodeling. On the other hand, when DARPP-32 is phosphorylated on Thr75, it enhances the β-adducin phosphorylation on Ser713 that modulates signaling in neurons and dendritic reaction facing rapid environment modifications [5,6,7].

Mutations in *ADD1, ADD2*, and *ADD3* genes have been associated with various diseases. In humans, variants rs4961 (G460W) and rs4963 (S586C) of *ADD1* are implicated in hypertension and cardiovascular disorders due to their effects on renal sodium handling [8,9]. Similarly, the rs564185858 (G367D) mutation in *ADD3* has been linked to neurodevelopmental disorders, including cerebral palsy and schizophrenia, while additional *ADD3* variants have been associated with biliary atresia and liver fibrosis [8,10]. Studies in animal models further support these findings, with mutations such as Q529R in rat ADDB being linked to hereditary spherocytosis and mutations in rat ADDA (F316Y) and ADDG (Q572R) influencing hypertension and renal dysfunction [8,9]. These variants compromise spectrin–actin cytoskeletal integrity, disrupt phosphorylation-dependent signaling pathways (PKC/PKA), and interfere with calcium–calmodulin binding, thereby contributing to disease mechanisms in conditions such as hypertension, neurodevelopmental disorders, and liver pathology. Although these associations are well established, the underlying molecular mechanisms by which adducin mutations contribute to disease remain to be fully understood [11].

Despite the known associations between adducin mutations and disease, the exact pathogenic mechanisms remain controversial. Some studies suggest that these mutations act as direct pathogenic drivers, while others propose their effects as epistatic ones, influenced by interactions with other genetic and environmental factors. For example, while G460W in ADDA has been widely studied for its potential role in hypertension, its impact varies across different populations, suggesting that additional modifiers may contribute to disease risk [11]. Similarly, the role of ADDB mutations in cancer remains debated, as it is unclear whether they act as driver mutations or arise as secondary effects of cytoskeletal dysregulation. These uncertainties underscore the need for alternative strategies to better characterize the functional impact of adducin variants.

In this context, recent computational studies highlight the utility of in silico approaches in identifying pathogenic mutations in cytoskeletal proteins. A study by Goswami [12] performed a comprehensive analysis of nonsynonymous SNPs in the *ADD2* gene and prioritized 27 deleterious variants based on multiple predictive tools. Several of these variants were associated with differential expression in cancers such as breast, colon, and pancreas, suggesting that cytoskeletal dysregulation driven by β-adducin mutations may contribute to tumor progression. Similarly, Kundu and Anand [13] investigated *ADD1* polymorphisms in the context of hypertension, identifying seven damaging nsSNPs, including the well-characterized G460W (rs4961), which is thought to disrupt renal ion transport and blood pressure regulation. These findings reinforce the link between adducin variants and both cardiovascular disease and cancer, underscoring the importance of further functional validation. Additionally, recent in silico analyses of *RB1* [14] and *TAGAP* [15] genes have demonstrated the impact of missense mutations on protein stability and function, further supporting the necessity of using multi-tool computational pipelines. Despite these advances, no study to date has systematically examined all three adducin isoforms using an integrated in silico strategy, despite their critical and conserved role in cytoskeletal regulation.

To address this gap, this study aims to systematically assess and prioritize high-risk mutations in *ADD1*, *ADD2*, and *ADD3* genes using an integrated computational framework. By leveraging predictive tools such as AlphaMissense (version 2023) [16], Rhapsody (version 2018) [17], PolyPhen-2 (version 2) [18], and PMut (version 2021) [19], we conducted in silico saturation mutagenesis to identify potentially pathogenic mutations. The most deleterious mutations were further analyzed in key regulatory regions, including phosphorylation sites and the calmodulin-binding domain. Additionally, structural impact assessments were performed using mCSM (version 2014) [20], DynaMut2 (version) [21], MutPred2 (version) [22], and Missense3D (version 2) [23], prioritizing mutations with high destabilization effects (prediction probability ≥ 0.8). Importantly, glycine substitutions emerged as the most destabilizing, likely due to their impact on backbone flexibility and local interaction networks. However, it remains unclear whether all destabilizing mutations directly result in pathogenicity or if some are functionally compensated within the cellular environment. This study provides a systematic approach for identifying pathogenic adducin mutations, guiding future experimental validation and therapeutic exploration.

## 2. Materials and Methods

### 2.1. Computational Prediction of Mutation Effects

This study analyzed the *ADD1*, *ADD2*, and *ADD3* genes, which encode the α-, β-, and γ-adducin proteins, respectively. In silico saturation mutagenesis was performed to assess the effect of all possible single amino acid substitutions across these genes. A multi-tool predictive approach was implemented by combining AlphaMissense [16], Rhapsody [17], PolyPhen-2 (HumDiv and HumVar) [18], and PMut [19]. This integrative strategy allowed for a more comprehensive classification of missense variants by encompassing structural, dynamic, evolutionary, and functional criteria (Table 1).

The selected tools offer complementary strengths: AlphaMissense employs deep learning trained on evolutionary and structural features, providing high precision for clinically relevant variants. Rhapsody incorporates protein dynamics and coevolutionary information. PolyPhen-2 is extensively validated for evaluating both rare and common variants. PMut is built upon a large and diverse mutation dataset, enhancing analysis robustness. Collectively, this strategy improves predictive power, supports prioritization of high-impact variants, and facilitates the selection of targets for experimental validation and therapeutic development. To prioritize the most deleterious missense mutations, we applied a stringent threshold of ≥0.8 probability score across all four predictive tools. This cutoff was chosen based on common practices in computational variant effect prediction, where a score closer to 1.0 typically reflects higher confidence in pathogenicity. By integrating only those variants predicted as pathogenic by all four tools at or above this threshold, we reduced the inclusion of borderline or uncertain cases and ensured that retained mutations represent consensus high-risk substitutions. This method increases the confidence in subsequent structural and functional interpretations, minimizing false positives and maximizing biological relevance.

### 2.2. Data Validation and Performance Evaluation

To validate the computational predictions, the identified mutations were cross-referenced with experimentally annotated variants from the UniProt database (version 2025_01) [24]. Each variant was categorized as a true positive, false positive, true negative, or false negative. Statistical performance metrics, including accuracy, sensitivity, precision, and F1-score, were computed. Cohen’s Kappa coefficient was also calculated to assess agreement between predicted and experimental classifications. All statistical analyses were conducted in Python (version 3.12.3) using the scikit-learn and pandas libraries.

### 2.3. Analysis in Specific Regions and Residues of Adducin Proteins

To detect mutations with the highest predicted pathogenicity at key functional sites, we focused on phosphorylation and calmodulin-binding regions, as annotated in UniProt for each adducin human protein: P35611 (ADDA), P35612 (ADDB), and Q9UEY8 (ADDG). Only mutations classified as pathogenic by all four predictive tools listed in Table 1 were considered for this focused regional analysis.

### 2.4. Stability Analysis of Pathogenic Missense Mutations

To further refine the selection of high-risk mutations, we applied a sequential filtering strategy based on predicted pathogenicity and structural impact. Only mutations predicted as pathogenic by all four tools, with a confidence score ≥ 0.8, were analyzed using a pipeline of four complementary programs: mCSM [20], DynaMut2 [21], MutPred2 [22], and Missense3D [23].

mCSM [20] uses graph-based structural signatures to estimate ΔΔG (change in Gibbs free energy) and assess protein stability. DynaMut2 [21] integrates normal mode analysis to evaluate conformational flexibility and thermal stability changes. MutPred2 [22] employs ensemble neural networks to predict both the likelihood of pathogenicity and the possible molecular mechanisms affected. Missense3D [23] evaluates structural damage through analysis of 3D geometry, identifying disruptions in hydrogen bonding, disulfide bridges, and steric constraints. This stepwise strategy enables precise prioritization of structurally and functionally disruptive mutations (Table 2).

**Table 2:** Information summary of structural and functional impact prediction tools.

Tool	Model Type	Underlying Data/Approach	Output Format	Score Interpretation	Website
mCSM [20]	Machine Learning (Regression)	Graph-based structural signatures and Gaussian process regression; trained on thermodynamic data	ΔΔG (change in Gibbs free energy)	Negative ΔΔG = destabilizing; positive ΔΔG = stabilizing	https://biosig.lab.uq.edu.au/mcsm (accessed on 18 January 2025)
DynaMut2 [21]	ML + Normal Mode Analysis	Combines graph-based structural features and NMA for flexibility analysis	ΔΔG and ΔTm (melting temperature change)	Negative ΔΔG = destabilizing; positive ΔΔG = stabilizing. Also evaluates changes in flexibility	https://biosig.lab.uq.edu.au/dynamut2 (accessed on 18 January 2025)
MutPred2 [22]	Neural Networks (Ensemble)	Trained on HGMD, SwissVar, and dbSNP; predicts functional and structural alterations	Pathogenicity score (0–1) + molecular mechanisms	Higher scores = higher likelihood of pathogenicity. Identifies potential mechanisms (e.g., loss of catalytic activity)	https://mutpred.mutdb.org (accessed on 18 January 2025)
Missense3D [23]	Structure-Based (non-ML)	Assesses 17 structural features via SCWRL4-based 3D modeling	Qualitative structural impact report	Classifies mutations as structurally damaging if they disrupt hydrogen bonds, disulfide bridges, or cause steric clashes	https://missense3d.bc.ic.ac.uk (accessed on 18 January 2025)

### 2.5. Protein–Protein Interaction Analysis

To investigate the structural context of adducin-mediated protein interactions, we used the cryo-EM structure 8IAH, which represents the spectrin–actin junctional complex from *Sus scrofa*. This model includes heterotetrameric adducin subunits in their native cytoskeletal assembly and was selected as the optimal structural reference [25]. To enable human-specific analysis, a homology-modeled humanized structure was generated using SWISS-MODEL(version 2018) [26] with 8IAH as the user-provided template. Only chains directly interacting with adducins were modeled: Chains 0, 1, 2, 3, 4, and 9 (adducin subunits) and Chains I, J, K, L, S, T, U, V, W, and X (interacting cytoskeletal proteins).

The resulting model was energy-minimized using GROMACS version 2023 [27] with the CHARMM36m (version 36) force field [28]. The system was solvated in a 15 nm cubic box with TIP3P water, neutralized with Na^+^ and Cl^−^ ions at 0.15 M, and minimized using steepest descent for 5000 steps with a convergence criterion of maximum force <1000 kJ/mol/nm. UCSF ChimeraX (version 1.9) [29] was used to identify residue–residue interactions, applying a distance cutoff ≤6 Å to define direct contacts.

**Figure 1:**
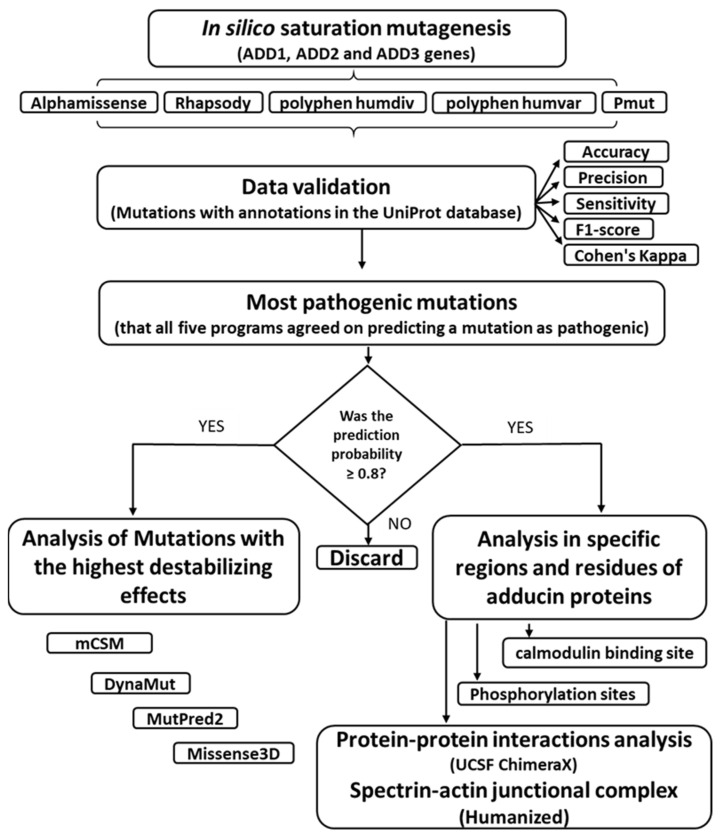
**Diagram of the methodological bioinformatics approach.** The workflow integrates multiple in silico tools to evaluate and prioritize pathogenic mutations in *ADD1, ADD2*, and *ADD3*. Each step supports a systematic analysis of structural, functional, and interaction-level consequences of the mutations.

Adducin residues involved in protein–protein interactions were then cross-referenced with the curated list of mutations predicted as pathogenic by all five tools. This comparative analysis aimed to identify mutations that could potentially disrupt structural and functional integrity within the spectrin–actin–adducin complex. The sequential steps for performing each analysis described in materials and methods are shown in Figure 1.

## 3. Results

### 3.1. In Silico Saturation Mutagenesis Analysis

**Figure 2:**
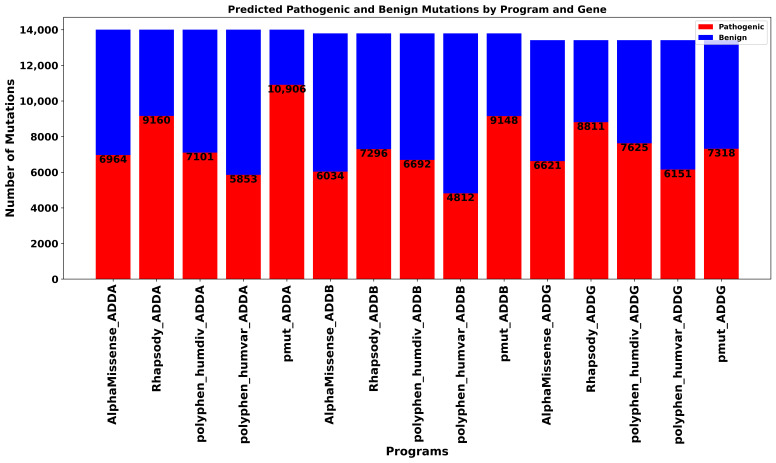
Distribution of predicted pathogenic and benign mutations for *ADD1*, *ADD2*, and *ADD3* genes across five predictive programs. The bar chart displays the number of mutations classified as pathogenic (red) and benign (blue) for each prediction tool. The analysis highlights variation in the predicted pathogenicity between programs and proteins.

We conducted an extensive in silico saturation mutagenesis analysis of the *ADD1*, *ADD2*, and *ADD3* genes, and of their products α-, β-, and γ-adducin proteins (ADDA, ADDB, and ADDG) to evaluate the potential functional impact of all possible missense mutations across their full-length sequences. In total, 14,003, 13,974, and 13,414 single amino acid substitutions were analyzed for ADDA, ADDB, and ADDG, respectively, using five widely recognized pathogenicity prediction tools: AlphaMissense [16], Rhapsody [17], PolyPhen-2 (HumDiv and HumVar models) [18], and PMut [19]. The results revealed substantial variability in pathogenicity predictions across the different algorithms and among the three adducin proteins (Figure 2).

For ADDA, PMut [19] predicted the highest proportion of pathogenic mutations (77.9%), suggesting a strong bias toward deleterious classification. In contrast, PolyPhen-2 (HumVar model) [18] was the most conservative, with only 41.8% of variants classified as pathogenic. Rhapsody [17] also showed a high proportion of pathogenic predictions (65.4%), while AlphaMissense (49.7%) [16] and PolyPhen-2 (HumDiv model, 50.7%) [18] produced more balanced outputs. In the case of ADDB, PMut [19] again identified the largest number of pathogenic variants (66.3%), followed by Rhapsody [17] (52.9%). AlphaMissense [16] predicted a slightly lower proportion of pathogenic mutations (43.7%), whereas PolyPhen-2 HumDiv and HumVar models [18] yielded more conservative estimates (48.5% and 34.9%, respectively). For ADDG, Rhapsody [17] exhibited the highest rate of predicted pathogenicity (65.7%), followed by PolyPhen-2 HumDiv (56.8%) [18] and PMut (54.5%) [19]. PolyPhen-2 HumVar (45.9%) [18] was again the most conservative, and AlphaMissense [16] showed nearly equal proportions of predicted pathogenic (49.4%) and benign (50.6%) mutations.

These findings underscore the heterogeneity in the output of different predictive algorithms and highlight the necessity of using a consensus-based or multi-tool approach. The observed discrepancies reinforce the importance of cross-validation strategies to improve the confidence and reproducibility of pathogenicity assessments in high-throughput mutational screenings.

We analyzed the most studied genetic variants that were reported in Uniprot, OMIM, and ClinVar for the three genes. For the *ADD1* gene, only variants Tyr270Asn (rs4971), Ala335Pro, Ser408Glu, Asn510Ile (rs4962) were present in the saturation mutagenesis analysis. For the *ADD2* gene, there were three amino acid changes in the positions that were studied before but none was as expected (rs4986, rs4982, rs17855969) and two that were not in the analysis (rs4987 and rs4985). Finally, for the *ADD3* gene, Gly367Asp (rs564185858) and Asn332Ser (rs41291894) variants that were previously analyzed were not present.

### 3.2. Evaluation of Each Model’s Performance

To assess the reliability of computational predictions, we compared them against curated experimental annotations from the UniProt database. Performance metrics including accuracy, precision, recall, F1-score, and Cohen’s Kappa were calculated for each model and protein.

Among all tools, PMut [19] exhibited the highest overall performance, particularly for ADDB (accuracy = 0.90, Kappa = 0.82) and ADDG (accuracy = 0.86, Kappa = 0.58). Rhapsody [17] performed best for ADDA (accuracy = 0.83, Kappa = 0.60), showing good concordance with experimentally validated variants.

PolyPhen-2 models [18] demonstrated moderate performance across proteins, with accuracies ranging from 0.63 to 0.81. Notably, AlphaMissense [16] performed poorly for ADDA (accuracy = 0.33, Kappa = −0.33), suggesting a significant discrepancy with the reference data. Similarly, Rhapsody [17] showed low agreement with UniProt annotations in ADDG (accuracy = 0.50, Kappa = 0.04). These findings confirm the importance of cross-validation and the complementary use of multiple predictors to improve accuracy and robustness in computational mutational analyses (Table 3).

PolyPhen-HumVar and PolyPhen-HumDiv [18] demonstrated moderate predictive reliability, with accuracies ranging from 0.63 to 0.81. In contrast, AlphaMissense [16] showed the poorest performance for ADDA (0.33 accuracy, −0.33 Kappa) and Rhapsody [17] was unreliable for ADDG (0.5 accuracy, 0.04 Kappa), suggesting poor agreement with validated data.

These findings highlight PMut [19] as the most reliable tool, particularly for ADDB and ADDG, while AlphaMissense [16] and Rhapsody (ADDG) [17] exhibit the lowest predictive power. The observed variability highlights the importance of cross-validation and points to the necessity of integrating multiple prediction tools to improve accuracy in pathogenicity assessments.

### 3.3. The Most Pathogenic Mutations in ADDA, ADDB, and ADDG Proteins

To identify the most pathogenic mutations in adducin proteins, we performed a consensus analysis using five predictive algorithms: AlphaMissense, Rhapsody, PolyPhen-2 (HumDiv and HumVar models), and PMut. Only missense variants predicted as pathogenic by all five programs were retained as having the highest pathogenic potential. This analysis yielded 4553 such variants in *ADD1* (ADDA), 3554 in *ADD2* (ADDB), and 3886 in *ADD3* (ADDG). These mutations are documented in Supplementary Table S1 adducin_mutations.xlsx, sheet: most_pathogenic_mutations.

**Figure 3:**
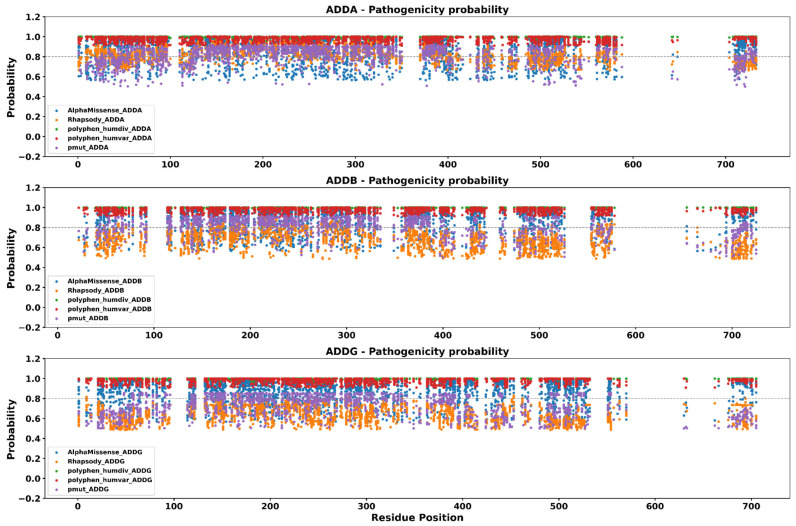
**Pathogenicity predictions of missense mutations across ADDA, ADDB, and ADDG proteins**. Each panel illustrates the prediction probability for every possible substitution as calculated by five widely used programs. Only mutations predicted as pathogenic by all tools are shown. The dashed horizontal line (0.8) represents the cutoff used to select mutations for structural analysis via mCSM, DynaMut2, MutPred2, and Missense3D.

To explore the patterns of distribution of predicted pathogenicity across the amino acid sequences, we applied a window-based approach to evaluate the regional distribution of high-confidence pathogenic variants. By using sliding windows of 10 amino acids, we quantified the density of consensus pathogenic mutations across each adducin isoform. To prioritize the most deleterious missense mutations, we applied a stringent threshold of ≥0.8 probability score across all four predictive tools as was described in materials and methods. The resulting profiles, shown in Figure 3, reveal distinct mutational cluster regions with a high concentration of predicted pathogenic substitutions, as well as extended gaps, corresponding to mutation-scarce or potentially tolerant segments of the protein sequences.

In ADDA, regions with the highest concentration of predicted pathogenic variants were identified at Residues 21–70, 121–160, 251–320, 371–400, 491–520, and 711–730. Notably, several of these regions overlap with experimentally validated phosphorylation sites (particularly S716 and S726) and include the calmodulin-binding domain spanning residues K717–K734. Conversely, a contiguous region from Residues 591–700 lacked any predicted pathogenic substitutions and coincides with an intrinsically disordered region (IDR), suggesting structural flexibility and tolerance to variation.

In ADDB, densely mutated segments were observed between Residues 41–60, 141–250, 281–320, 361–390, 481–520, and 701–720. Among these, critical phosphorylation sites such as T55, S703, and S713 were located in mutational hotspots, as well as the calmodulin-binding domain encompassing K704–K721. Meanwhile, Residues 581–650 formed a long gap without predicted pathogenic mutations, which also corresponds to a predicted IDR.

For ADDG, Regions 41–70, 131–300, 381–410, 491–530, and 681–700 exhibited the greatest density of consensus pathogenic substitutions. These segments include experimentally supported phosphorylation targets such as S64, S442, and Y446, as well as the calmodulin-binding region (K684–K688). In contrast, Residues 571–660 represent an extended benign region with no high-confidence pathogenic predictions, again mapping to an IDR.

These findings underscore the uneven landscape of mutational vulnerability in adducin proteins. Regions enriched in phosphorylation or calmodulin-binding sites tend to harbor the highest concentration of predicted deleterious variants, while structurally disordered or less constrained segments appear more mutation-tolerant. These insights may inform future functional studies and help prioritize regions for experimental validation.

### 3.4. Mutations with the Highest Impact in Key Domains

The analysis of mutations within functionally relevant domains or conserved regions is essential to predict their potential structural and functional consequences. In adducin proteins, key functional elements include phosphorylation sites regulated by kinases such as PKC and PKA, as well as calmodulin-binding regions. Variants affecting these sites may alter the protein’s conformational state, disrupt signaling pathways, and impair cytoskeletal organization. Therefore, we evaluated the spatial distribution of high-impact mutations within these specific regions to better understand their potential pathogenic effects.

#### 3.4.1. Mutations That Affect Phosphorylation Sites

To determine the impact of pathogenic mutations in functionally relevant regions, we analyzed their distribution in phosphorylation and calmodulin-binding sites. Among the 741 annotated phosphorylation-related substitutions in ADDA, 120 (16.2%) were consistently predicted as deleterious by all five programs. In ADDB, only 58 out of 551 substitutions (10.5%) and 88 out of 589 (14.9%) phosphorylation-site substitutions in ADDG passed the pathogenicity threshold. These findings indicate a notable vulnerability of phosphorylation sites in ADDA compared to the other adducin proteins.

Serine (S) and Tyrosine (Y) residues were the most frequently mutated amino acids across all three proteins. In ADDA, S64, Y24, and Y35 exhibited the highest number of substitutions. Similarly, in ADDB, S60, S701, and S713 were the most frequently altered sites. For ADDG, S64, S442, and Y446 showed the greatest variation in mutational profiles. These residues are likely critical for phosphorylation-mediated regulation, suggesting that mutations at these sites may significantly impact protein function.

In terms of substitution patterns, serine-to-alanine (S→A), serine-to-threonine (S→T), and tyrosine-to-phenylalanine (Y→F) mutations were among the most common. The high frequency of S→A substitutions suggests a potential disruption of phosphorylation sites, as alanine lacks the hydroxyl group required for phosphorylation. Additionally, tyrosine-to-phenylalanine mutations are particularly relevant, as both residues share a similar structure, but phenylalanine cannot undergo phosphorylation, potentially affecting signaling pathways. These findings highlight the vulnerability of phosphorylation sites to pathogenic mutations and suggest that certain residues, such as S64 in both ADDA and ADDG, and S713 in ADDB, may play crucial roles in protein stability and function. All mutations affecting phosphorylated residues are shown in the Supplementary Table S1 adducin_mutations.xlsx; sheet: phosphorylated residues.

#### 3.4.2. Mutations That Affect Calmodulin-Binding Sites

A total of 341, 342, and 342 possible mutations were analyzed for calmodulin-binding sites in ADDA, ADDB, and ADDG proteins, respectively. Among these, 188 (55.1%) in ADDA, 138 (40.3%) in ADDB, and 136 (39.8%) in ADDG were predicted to be pathogenic mutations. Lysine (K) and Phenylalanine (F) residues were the most frequently mutated amino acids across all three proteins. In ADDA, K716, K717, and K718 exhibited the highest number of substitutions. Similarly, in ADDB, K704, K705, and K706 were the most frequently altered sites. For ADDG, K684, K685, and K688 showed the greatest variation in mutational profiles. These residues are crucial for calmodulin interaction, suggesting that mutations at these positions may significantly impact protein function and regulatory mechanisms.

In terms of substitution patterns, lysine-to-glutamate (K→E), lysine-to-glutamine (K→Q), and phenylalanine-to-tyrosine (F→Y) mutations were among the most common. The high frequency of K→E substitutions suggest a potential disruption of calmodulin binding, as glutamate introduces a negative charge, altering electrostatic interactions. Similarly, phenylalanine-to-tyrosine mutations may modify hydrophobic interactions, possibly affecting the protein’s ability to bind calmodulin effectively.

These findings highlight the sensitivity of calmodulin-binding sites to pathogenic mutations and suggest that residues such as K716 in ADDA, K704 in ADDB, and K684 in ADDG may play essential roles in protein function. Further biochemical and structural analyses are required to determine the precise impact of these mutations on calmodulin binding and downstream signaling events. All mutations affecting the *calmodulin binding region* are shown in the Supplementary Table S1 adducin_mutations.xlsx sheet: calmodulin_binding_region.

### 3.5. Mutations with the Highest Destabilizing Effects

The sequential analysis of mutations in the ADDA, ADDB, and ADDG proteins using mCSM [20], DynaMut2 [21], MutPred2 [22], and Missense3D [23] allowed for a progressive refinement of potentially destabilizing mutations analysis. The filtering process ensured that only mutations with the highest likelihood of compromising structural integrity were retained for further evaluation. All the most destabilizing mutations are shown in the Supplementary Table S1 adducin_mutations.xlsx sheet: most_destabilizing_mutations.

For ADDA, 15.64% of mutations (2190/14,000) met the probability threshold (≥0.8), and 350 from 2190 mutations (15.98%) were classified as highly destabilizing by mCSM [20]. Within this subset, 73 from 350 mutations (20.86%) were confirmed by DynaMut2 [21] to have a predicted stability change (ΔΔG^stability^) ≤ −3.0 kcal/mol. Further filtering using MutPred2 [22] identified 18 from 73 mutations (24.66%) as causing altered stability, of which 12 were validated by Missense3D [23] as structurally damaging.

For ADDB, 3.74% of mutations (523) exceeded the probability threshold (≥0.8). From these, 156 mutations (29.83%) were identified as highly destabilizing by mCSM [20]. DynaMut2 [21] confirmed 43 from 156 mutations (27.56%) with a stability change of ≤ −3.0 kcal/mol. Of this subset, 5 from 43 mutations (11.63%) were predicted by MutPred2 [22] to alter stability, and 3 of these 5 mutations were further validated by Missense3D [23] to cause structural damage.

For ADDG, only 0.22% of mutations (29) met the probability threshold (≥0.8). Among these, 8 mutations from 29 (27.59%) were flagged as highly destabilizing by mCSM [20]. However, none of these mutations met the DynaMut2 [21] threshold, and consequently, no mutations from ADD3 progressed to MutPred2 [22] or Missense3D [23] analysis.

These results indicate that ADD1 harbored the highest proportion of destabilizing mutations, followed by ADD2, while ADD3 exhibited very few mutations predicted to significantly affect structural stability.

**Figure 4:**
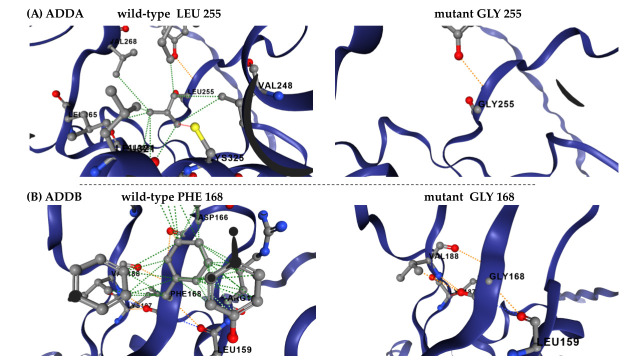
**DynaMut2 prediction of molecular flexibility and structural destabilization effect of ADDs mutations.** (**A**) Leu255Gly has a predicted destabilizing effect (ΔΔGstability of −3.01 kcal/mol), modelled in ADDA. (**B**) Phe168Gly has a predicted destabilizing effect (ΔΔGstability of −3.2 kcal/mol), modelled in ADDB.

The most destabilizing mutations, consistently identified across all prediction tools, affected leucine (L), isoleucine (I), and valine (V) residues, particularly in ADDA. In this case, the amino acids L197, I298 and L299 were among the most frequently altered positions. In ADDB, L274, F168, and I170 were the most affected residues, with glycine (G) substitutions being predominant. The prevalence of leucine and isoleucine substitutions suggests potential disruptions in hydrophobic core stability, which could significantly impact protein folding and function. The example of changes in the network of interactions between the wild-type residue and the surrounding residues is shown in Figure 4.

### 3.6. Adducins–Proteins Interaction Analysis

The quality of the humanized structural model generated via SWISS-MODEL [26] was evaluated using standard validation metrics. The model achieved a QMEANDisCo global score of 0.76 ± 0.05, indicating good overall geometrical quality suitable for structural analyses. Although the GMQE score was –1.70 (reflecting potential limitations in sequence alignment coverage or model confidence), the model exhibited excellent structural conservation, with a root mean square deviation (RMSD) of 0.348 Å compared to the original template. Subsequent energy minimization using GROMACS [27] resolved steric clashes and refined the geometry. Visual inspection and Ramachandran plot analysis confirmed structural integrity, with over 93% of residues located in favored or allowed regions. Together, these data support the reliability of the model for downstream residue-level interaction analysis.

**Figure 5:**
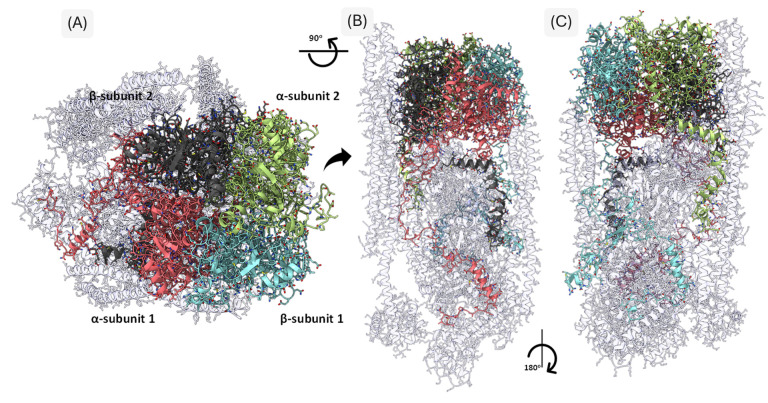
**Three-Dimensional Representation of the Humanized Cytoskeletal Protein Complex.** The figure illustrates axial (**A**) and lateral (**B**,**C**) views of the humanized model derived from PDB entry 8IAH. The adducin tetramer is color-coded: α-subunit 1 in pink, α-subunit 2 in light green, β-subunit 1 in light blue, and β-subunit 2 in dark gray. Interacting cytoskeletal proteins are shown in light gray, highlighting the extensive network of inter-subunit and inter-protein contacts.

Analysis of residue–residue contacts within the humanized spectrin–actin–adducin complex revealed an extensive interaction network between adducins and other cytoskeletal components (Figure 5). A total of 2023 residues were identified as participating in protein–protein interfaces, from which 800 residues corresponded to direct contacts between adducin subunits and neighboring proteins that include 190 in α-subunit 1 (chain G), 175 in α-subunit 2 (chains N and M), 212 in β-subunit 1 (chain H), and 223 in β-subunit 2 (chains K and L). Additionally, 643 residues belonged to interacting regions in non-adducin proteins: actin (chains A, B, C, P), tropomyosin (chains E, F, I, J), and spectrin β (chains O and D).

Interactions between adducin subunits themselves, essential for tetramer formation, involved 589 residues. This oligomerization is critical for their structural and regulatory functions within the spectrin–actin cytoskeleton. Mapping these contacts provides a structural framework for assessing the impact of mutations on protein–protein interactions and the stability of the junctional complex. A full list of interface residues is available in the Supplementary Table S1 adducin_mutations.xlsx, sheet “contact_residues”.

Residue frequency analysis revealed characteristic interaction profiles for α- and β-adducin subunits. In α-subunits, leucine (L) was the most abundant interface residue (28 occurrences), followed by alanine (A, 17), glutamic acid (E, 16), glycine (G, 13), and arginine (R, 11). β-subunits exhibited a similar trend: leucine (25), alanine (21), glutamic acid (18), glycine (15), and arginine (16) were the most frequently observed. These amino acids possess physicochemical properties conducive to protein–protein interface stabilization: hydrophobic residues like leucine and alanine contribute to core packing and to the structural integrity on interface, while charged residues such as glutamic acid and arginine likely participate in electrostatic interactions and hydrogen bonding, Glycine, due to its minimal side chain, may promote conformational flexibility at tight turns or interface edges.

Subtle differences in residue distribution suggest subunit-specific roles during protein–protein interactions. Cysteine was notably absent from all interface residues, indicating disulfide bonding does not contribute to adducin interactions in this context. Instead, interaction interfaces appear to be stabilized by a combination of hydrophobic forces, polar interactions, and electrostatic complementarity.

**Figure 6:**
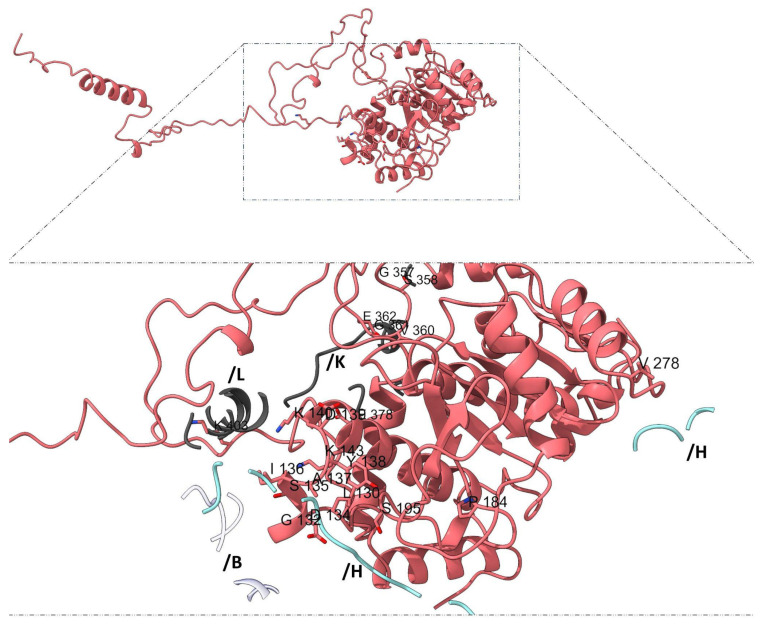
**Unique interaction residues in α-subunit 1 (chain G).** Highlighted residues from the chain G (pink) participate exclusively in interactions with β-adducin subunit 2 (light gray, chains K, L), b-adducin subunit 1 (light blue, chain H), and actin (white, chain B). In α-subunit 1, the interaction interface comprises residues such as L130, G132, D134, S135, I136, A137, Y138, D139, K140, K143, P184, S195, V278, G357, S358, V360, G361, E362, E378, and K403.

**Figure 7:**
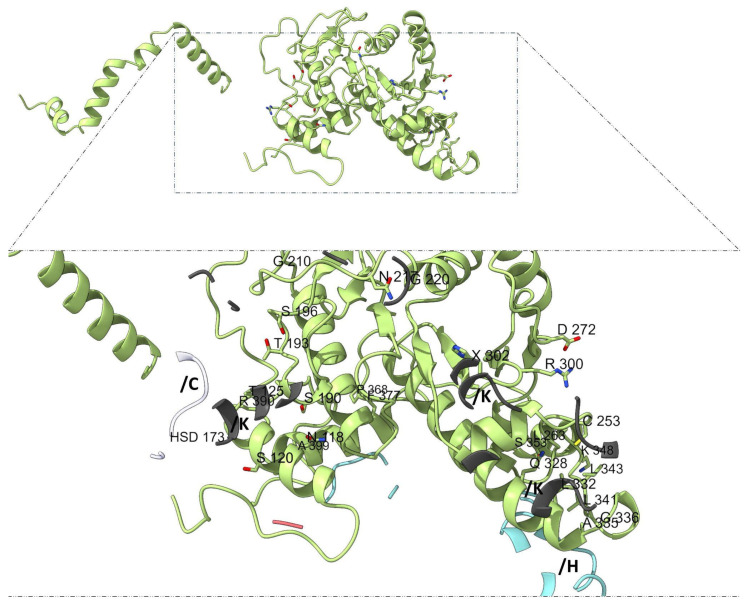
**Interaction interface of α-subunit 2 (chains N and M).** Distinct residues from chains N and M (light green) engaged in subunit-specific contacts are visualized relative to neighboring proteins as actin (white, chain C) and β-adducin subunit 1 (light blue, chain H) and subunit 2 (dark gray, chain K). α-subunit 2 displays a distinct set of residues, including N118, S120, T125, S190, T193, G210, and C253.

Mapping subunit-specific contact residues further supports the notion of structural and functional asymmetry among adducins. In α-subunit 1 (chain G), the interaction interface includes residues such as L130, G132, D134, S135, I136, A137, Y138, D139, K140, and K143, among others (Figure 6), whereas α-subunit 2 (chains N and M) displays a distinct set of residues, including N118, S120, T125, S190, T193, G210, and C253 (Figure 7).

This non-redundant distribution of interface residues between homologous subunits suggests that the adducin tetramer does not operate as a symmetrical or mirror assembly. Instead, each subunit contributes unique molecular interactions that likely reflect specialized structural roles or regulatory functions. This asymmetric contact landscape is essential for identifying subunit-specific determinants of stability, interaction affinity, or post-translational regulation. These insights provide a framework for investigating how particular mutations—especially those affecting residues unique to one subunit—could selectively disrupt tetramerization or impair specific protein–protein interactions within the cytoskeletal complex.

**Figure 8:**
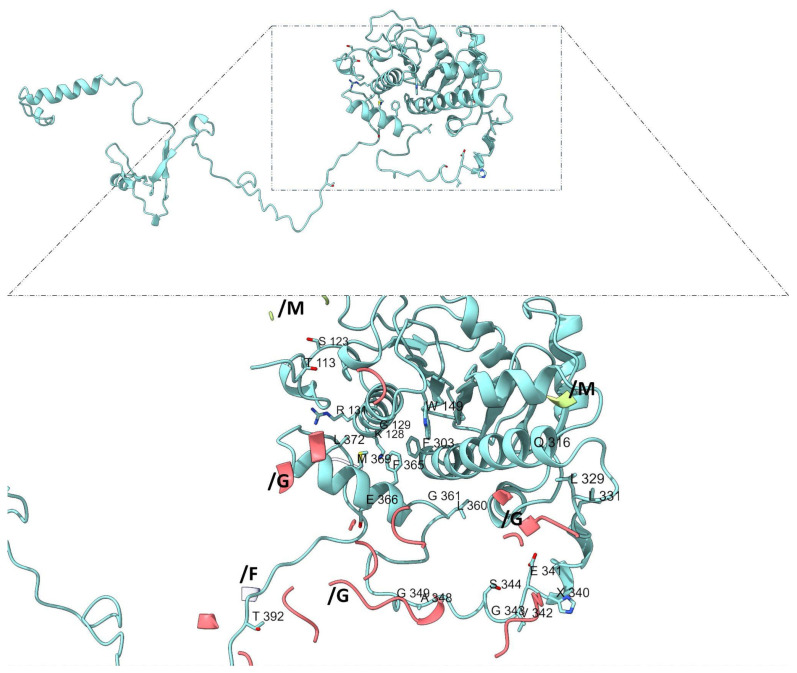
**Interaction sites in β-adducin subunit 1 (chain H).** Residues from chain H (light blue) are labeled and shown within their spatial context in the protein–protein interactions with α-adducin subunit 1 (pink, chain G), with topomyosin (white, chain F) and α-adducin subunit 2 (ligth green, chain M). b-adducin subunit 1 includes unique interaction residues as T113, S123, K128, G129, R131, W149, and F303.

**Figure 9:**
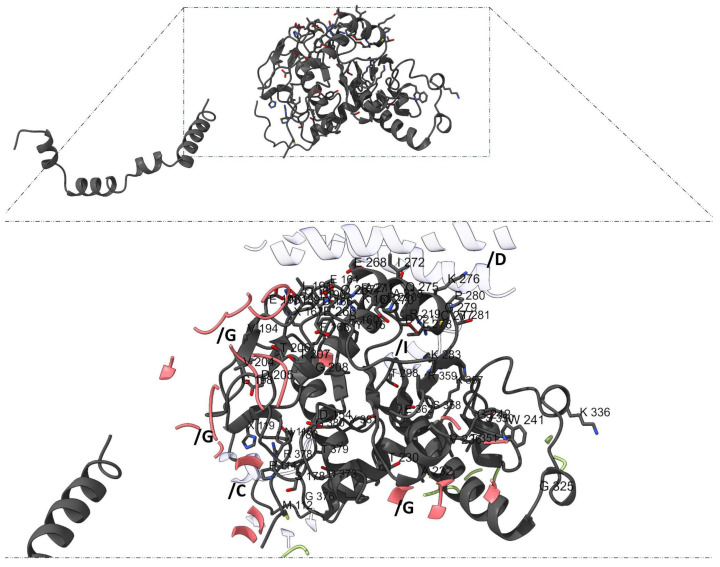
**Interaction pattern in β-adducin subunit 2 (chains K and L).** The amino acids in chains K and L (dark gray) exhibit the highest diversity of unique interaction residues, in protein–protein arrangement interactions with α-adducin subunit 1 (chain G), actin (chain C), and spectrin (chain D). β-subunit 2 contains the highest number of distinct interaction residues, including M112, P114, R160, F168, D205, G279, K336, and R378 some of which are legible in the image.

Among β-subunits, β-subunit 1 (chain H) includes unique interaction residues such as T113, S123, K128, G129, R131, W149, and F303 (Figure 8), while β-subunit 2 (chains K and L) contains the highest number of distinct contact residues (e.g., M112, P114, R160, F168, D205, G279, K336, and R378) (Figure 9).

To identify structurally and functionally constrained residues within adducin, we leveraged our saturation mutagenesis dataset to evaluate the mutational tolerance of amino acid positions involved in protein–protein interactions. In this analysis, each residue can theoretically be substituted by 19 other amino acids. When the vast majority of these substitutions are predicted to be pathogenic, the residue is considered functionally constrained—a hallmark of positions essential for maintaining structural integrity, biochemical function, or specific intermolecular interactions. We applied a high-stringency threshold: residues were classified as constrained if ≥17 out of 19 substitutions (89% or more) were predicted as deleterious by all four algorithms. This cutoff is consistent with thresholds used in deep mutational scanning studies and offers a strong indicator of structural or evolutionary intolerance to amino acid change.

In α-adducin subunit 1 (chain G), 137 of the 190 interface residues had matching entries in the mutagenesis dataset (ADD1). Among these, three residues—K140 and K143 (each with 18 predicted pathogenic substitutions), as well as P184 (17/19)—emerged as particularly constrained. Their intolerance to substitution suggests a critical role in maintaining subunit interactions or overall structural stability. In α-subunit 2 (chains N and M), 239 interface residues were mapped. Only three failed to meet consensus pathogenic predictions, while four residues surpassed the high-pathogenicity threshold: G210 (19/19), R300 (18/19), H302 (17/19), and R390 (17/19). The full intolerance of G210 to any substitution highlights its likely irreplaceable structural or functional role. The β-adducin subunit 1 (chain H) exhibited 155 matches in the ADD2 dataset. While most residues displayed moderate tolerance, two positions—W149 (18/19) and G361 (17/19)—met the high-stringency criteria, suggesting critical involvement in structural contacts or flexibility.

Strikingly, β-subunit 2 (chains K and L) harbored the largest number of constrained positions. Of 509 matched interface residues, 12 surpassed the ≥17 substitution threshold: R160 (18), G198 (17), R219 (18), P220 (19), D221 (19), V236 (18), G242 (19), G279 (19), D373 (18), G376 (19), T379 (18), and G380 (19). These residues likely serve as mutational bottlenecks—sites at which any amino acid replacement compromises protein stability or interaction specificity. Notably, five residues (P220, D221, G242, G279, G376) were completely intolerant to variation, marking them as potential structural anchors or critical contact points.

This pattern of residue-level intolerance not only underscores the essential contribution of these amino acids to the assembly and function of the adducin tetramer, but also supports the broader hypothesis of structural and functional asymmetry among subunits. Mapping such constrained residues offers valuable targets for future biochemical studies, particularly in understanding disease-associated mutations or in guiding rational mutagenesis experiments.

## 4. Discussion

This study systematically evaluated the pathogenic and structural consequences of all possible missense mutations in the *ADD1*, *ADD2*, and *ADD3* genes using an integrative computational saturation mutagenesis strategy. By combining multiple predictive tools, we refined the classification of missense variant effects on pathogenicity, protein stability, and structural integrity. Notably, the predictions showed substantial variability across tools, underscoring the importance of a multi-tool approach to obtain a robust and consensus-based assessment of mutational impact. For instance, PMut [19] tended to classify a greater number of variants as pathogenic, particularly in *ADD1* and *ADD2* genes, whereas PolyPhen-2 [18] was more conservative in its predictions, likely due to differences in their algorithmic frameworks, training datasets, and sensitivity to sequence-structural context.

A comparison of our findings with previous studies highlights both the broader scope and the increased resolution of our approach. Goswami et al. [12] investigated nonsynonymous SNPs (nsSNPs) in ADD2 and identified 27 pathogenic variants associated with cancer. Similarly, Kundu and Anand [13] evaluated nine nsSNPs in ADD1 and identified G460W (rs4961) as structurally destabilizing and associated with hypertension. Their work focused on variants of known clinical relevance and involved detailed modeling of individual substitutions. In contrast, our saturation mutagenesis approach covered the entire coding regions of the three genes, yielding a more comprehensive landscape of mutational vulnerability: 4553, 3554, and 3886 predicted pathogenic substitutions in ADD1, ADD2, and ADD3, respectively, based on consensus predictions from four tools.

This multi-tool strategy has been successfully applied in other studies investigating genes such as TP53 [30], CFTR [31], and BRCA1/2 [32], particularly in the context of deep mutational scanning and computational variant effect prediction. In our case, this approach enabled the identification of regions with a high local density of predicted pathogenic mutations across the ADDA, ADDB, and ADDG isoforms. These mutation-enriched clusters—detected using a sliding window analysis of consensus predictions—coincide with functionally important domains, including phosphorylation sites and calmodulin-binding regions, underscoring their structural and regulatory significance. In contrast, other segments of the protein sequences were almost entirely devoid of pathogenic substitutions. These mutation-scarce regions aligned with predicted intrinsically disordered regions (IDRs), suggesting that local structural flexibility may confer a degree of mutational tolerance. This spatial distribution of mutational impact reveals a non-uniform vulnerability landscape across the adducin proteins, offering insights into site-specific functional constraints. By integrating this comprehensive in silico dataset with protein structural models and known interaction sites, our analysis offers a high-resolution mutational atlas for the adducin family that could inform future experimental studies and disease variant prioritization.

The analysis of intrinsically disordered regions (IDRs) further supports the idea that these regions may be functionally resilient to mutations. IDRs facilitate protein–protein interactions but are difficult to assess using conventional structure-based methods. Recent studies indicate that predicting mutation effects in IDRs remains a challenge due to their conformational heterogeneity and lack of stable secondary structures [33]. These regions often display high sequence variability, which may contribute to functional redundancy and compensatory mechanisms that mitigate the impact of mutations. Based on the UniProt database, the disordered regions that are shared between adducins A, B, and G (α-, β-, γ-) are the N-terminal regions (amino acids 1–20, 1–25, and 1–20, respectively) and the MARCKS domain (amino acids 717–734, 704–721, and 684–710, respectively). Although the major IDRs are located within the C-terminal coil domain, their boundaries differ between isoforms: 421–486 in ADDA, 430–480 in ADDB, and 471–497, 535–555, and 575–610 in ADDG. Furthermore, variant Gly460Trp (rs4961) in ADD1, linked to arterial hypertension [8], and G367D (rs564185858) in ADDG, associated with cerebral palsy [34], represent compelling cases where mutations in an IDR (ADDA) or in a critical neck region (ADDG), and they have been implicated in disease despite not being predicted as destabilizing in our computational analysis. The rs4961 variant may alter dynamic interaction networks, while rs564185858 has been shown to disrupt neuronal cell migration, proliferation, and actin cytoskeleton regulation [34,35]. These findings highlight a key limitation of current computational tools, which may overlook non-structural functional consequences of mutations, especially in the context of IDRs.

Despite these advances, the functional consequences of adducin mutations remain a topic of debate. Some evidence suggests that certain variants act as direct disease drivers, while others may exert epistatic effects modulated by genetic background or environmental factors. For example, the impact of the G460W mutation in ADD1 gene appears to differ across populations, implicating the influence of modifier loci. Similarly, while ADD2 gene mutations have been linked to tumor progression, it remains unclear whether they serve as primary oncogenic drivers or act indirectly by disrupting cytoskeletal dynamics. Our analysis also confirms that serine and tyrosine residues within phosphorylation sites are among the most frequently mutated, supporting previous observations by Goswami et al. on the importance of post-translational regulation in ADD2-related cancer [12].

Furthermore, phosphorylation data from UniProt and experimental studies highlight specific sites in adducins with confirmed kinase associations [5,36]. In ADDA, residues S59, S408, S436, and S481 are phosphorylated by PKA, while T445 and T480 are substrates of ROCK2; S716 is targeted by PKC, and S726 by both PKA and PKC. In ADDB, T55 is phosphorylated by PKA, S703 by PKC, and S713 by both PKA and PKC. These phosphorylation events are central to regulating the dynamic association of adducins with the spectrin–actin cytoskeleton, modulating membrane stability, cell shape, and motility. Experimental evidence has shown that phosphorylation by PKA or PKC leads to dissociation of adducin from spectrin–actin complexes, thereby promoting cytoskeletal remodeling and increased cell migration. Conversely, inhibition or mutation of these phosphosites leads to persistent membrane anchoring, impaired lamellipodia formation, and defective cell motility.

Notably, among all experimentally supported phosphorylation sites, only T480 and T481 in ADDA were not predicted to be pathogenic by the four consensus tools, suggesting that these residues may be functionally modulated without inducing structural destabilization. In contrast, phosphosites such as S716 and S726 in ADDA and S703 and S713 in ADDB, which are located within the calmodulin-binding domain, were predicted as highly pathogenic. These sites play a critical role in calcium-dependent regulation of adducin function. Calmodulin binding is known to mask phosphorylation sites and modulate sensitivity to kinases, linking calcium signaling with cytoskeletal regulation [5]. Disruption of these regulatory nodes by pathogenic mutations could uncouple adducin from upstream signals, leading to aberrant actin dynamics, membrane instability, or altered cell polarity—phenotypes associated with vascular dysfunction, hypertension, and tumor cell invasion [5,36]. These findings underscore the relevance of phosphorylation sites not only as structural features but also as functional switches controlling adducin activity and cellular behavior. Their accurate annotation in mutational impact models is thus essential for understanding disease mechanisms.

Importantly, we incorporated multiple structure-based tools (mCSM [24], DynaMut2 [20], MutPred2 [21], and Missense3D [22]) to identify the most destabilizing substitutions. This approach revealed that leucine (L), isoleucine (I), and valine (V) residues are particularly vulnerable to destabilization—an aspect underrepresented in earlier studies based on single predictors. Notably, substitutions involving glycine (G) also stood out as highly destabilizing, likely due to its small side chain and role in maintaining local flexibility. These findings highlight the structural sensitivity of both bulky hydrophobic residues and glycine in key regions of the adducin proteins.

The impact of these substitutions is further clarified through the ΔΔG values (the change in Gibbs free energy between wild-type and mutant structures), which serve as an indicator of the mutation-induced change in protein stability. A significantly negative ΔΔG value typically reflects a destabilizing effect, potentially leading to partial unfolding, local misfolding, or altered conformational dynamics that impair the protein’s ability to maintain its structure and interact with partners. In cytoskeletal scaffolding proteins like adducins, even subtle destabilization can compromise the formation of stable heterotetramers or disrupt interactions with actin filaments, spectrin, or regulatory molecules such as calmodulin. Moreover, destabilized regions may become more susceptible to proteolytic degradation or fail to respond appropriately to post-translational modifications like phosphorylation, which are essential for activity regulation and cellular localization. Therefore, ΔΔG is not merely a structural metric: it is a biologically relevant parameter that links molecular alterations to potential loss-of-function phenotypes and disease mechanisms.

Comparative analysis with studies on Rb1 [14] and TAGAP [15] revealed consistent trends in how pathogenic mutations affect protein stability. In all three proteins, PMut identified the highest number of damaging variants, while tools like PROVEAN [37] produced more conservative classifications. In the ADD genes, pathogenic variants were most concentrated in phosphorylation and calmodulin-binding regions, while in Rb1, destabilization was associated with mutations in structurally constrained domains. The TAGAP study similarly highlighted variants affecting flexible regulatory elements. Together, these findings suggest that the structural context—whether rigid or dynamic—strongly influences mutational impact. Across all proteins analyzed, glycine substitutions consistently emerged as particularly disruptive, reinforcing their role in altering backbone flexibility and destabilizing local interaction networks.

Our findings underline the significant role of glycine substitutions in altering conformational flexibility and disrupting local interaction networks. Despite glycine’s minimal side chain, which imparts high flexibility to protein backbones, our results indicate that nonpolar-to-glycine substitutions were among the most destabilizing ones. Glycine can disrupt the stability and dynamics of secondary structure interactions, particularly at α-helix/β-sheet and helix-helix interfaces, leading to pronounced destabilization. While previous studies have reported stabilizing effects of glycine substitutions in β-turn sequences [38] and active sites [39], the impact of glycine substitutions remains highly context-dependent. While glycine may stabilize certain structural motifs, its incorporation in other structural elements can lead to destabilization, as observed in this study.

Furthermore, our findings are consistent with previous experimental studies showing the functional relevance of specific residues at protein-protein interfaces. For example, research on the c-NADP-malic enzyme demonstrated that point mutations in residues located at dimer or tetramer interfaces affect both the stability and enzymatic activity of the complex [40]. Similarly, mutational analyses of the AXH tetramer revealed that altering interface residues leads to a destabilization of the oligomeric state, confirming their importance for structural integrity [41]. Thus, theoretical and computational studies have supported the idea that the spatial arrangement and properties of side chains at the interface are key determinants of protein folding and interaction specificity.

One of the main limitations of this study is that the analysis was not conducted through experimental approaches or molecular dynamics simulations, which would have provided additional validation and insights into the stability and temporal evolution of the protein-protein interactions identified. The data were derived from a static three-dimensional structure, which does not capture the conformational flexibility or dynamic behavior of the adducin tetramer under physiological conditions. Despite these limitations, the study offers a valuable perspective by identifying key residues at the protein-protein interfaces and highlighting differences and similarities between α and β subunits. The integration of structural data with pathogenic mutation analysis strengthens the relevance of the findings and provides a foundation for future experimental validation. As a future direction, molecular dynamics simulations and site-directed mutagenesis could be employed to further investigate the functional roles of these interface residues and to assess the impact of specific mutations on the structural integrity and interaction network of the adducin complex.

## 5. Conclusions

This study provides a systematic in silico assessment of all possible missense mutations in the *ADD1*, *ADD2*, and *ADD3* genes, revealing key structural and functional consequences. The results underscore the value of using multiple predictive tools, as PMut demonstrated higher sensitivity in identifying pathogenic variants, whereas PolyPhen-2 offered more conservative predictions. Structure-informed methods, particularly Rhapsody, proved especially informative in evaluating constrained or functionally critical regions.

Among the findings, glycine substitutions consistently resulted in strong destabilizing effects, emphasizing the importance of residue-specific and context-dependent analyses. Although glycine plays functional roles in flexible regions, its substitution in structured domains can significantly compromise protein stability.

These findings support the integration of computational mutagenesis with structural modeling to identify high-risk variants in cytoskeletal proteins. However, experimental validation is essential to confirm the pathogenic potential of prioritized mutations. Future efforts should focus on combining computational and experimental data to improve the precision of pathogenicity prediction and guide therapeutic exploration for adducin-related diseases.

## Figures and Tables

**Table 1:** Information summary of missense pathogenicity prediction tools.

Tool	Model Type	Training Data/Features	Output Format	Score Interpretation	Website
AlphaMisse nse [16]	Machine Learning	Integrates protein structural data and evolutionary constraints; validated with ClinVar	Score from 0 to 1	Higher scores indicate greater pathogenicity. Achieves 90% precision; classifies 32% as pathogenic and 57% as benign.	https://alphamissense.hegelab.org(accessed on 7 January 2025)
Rhapsody [17]	Random Forest	Trained on 87,726 ClinVar and UniProt variants; uses conservation, coevolution, and dynamics	Score from 0 to 1	Higher scores indicate stronger deleterious effects.	http://rhapsody.csb.pitt.edu/sat_mutagen.php (accessed on 7 January 2025)
PolyPhen-2 [18]	Naïve Bayes	Annotated human variants; structural modeling and evolutionary conservation; HumDiv and HumVar datasets	Score from 0 to 1 with qualitative classification (benign, possibly damaging, probably damaging)	Higher scores indicate greater likelihood of functional damage. Different sensitivity based on selected dataset.	http://genetics.bwh.harvard.edu/pph2/ (accessed on 7 January 2025)
Pmut [19]	Random Forest	Trained on 65,000 mutations from 12,141 proteins; includes conservation, physicochemical, and interactome features	Score from 0 to 1	Scores ≥ 0.5 indicate pathogenic mutations.	http://mmb.irbbarcelona.org/PMut/predictor/new/ (accessed on 7 January 2025)

**Table 3:** The performance metrics of prediction programs for the ADDA, ADDB, and ADDG proteins.

Program_protein	Accuracy	Precision	Recall	F1-Score	Cohen’s Kappa
**ADDA** AlphaMissense_ADDA	0.33	0	0	0	−0.33
**Rhapsody_ADDA**	**0.83**	**0.83**	**0.83**	**0.83**	**0.6**
polyphen_humdiv_ADDA	0.66	0.625	0.83	0.71	0.33
polyphen_humvar_ADDA	0.75	0.8	0.66	0.72	0.5
pmut_ADDA	0.58	0.55	0.83	0.66	0.17
**ADDB** AlphaMissense_ADDB	0.81	1	0.66	0.8	0.65
Rhapsody_ADDB	0.81	1	0.66	0.8	0.65
polyphen_humdiv_ADDB	0.72	0.8	0.66	0.72	0.46
polyphen_humvar_ADDB	0.81	1	0.66	0.8	0.66
**pmut_ADDB**	**0.90**	**1**	**0.83**	**0.9**	**0.82**
**ADDG** AlphaMissense_ADDG	0.77	0.5	0.4	0.44	0.3
Rhapsody_ADDG	0.5	0.25	0.6	0.35	0.04
polyphen_humdiv_ADDG	0.63	0.28	0.4	0.33	0.09
polyphen_humvar_ADDG	0.72	0.4	0.4	0.4	0.22
**pmut_ADDG**	**0.86**	**0.75**	**0.6**	**0.66**	**0.58**

Bold letters indicate the programs with the best metrics for each gene.

## Data Availability

The original contributions presented in this study are included in the article/Supplementary Materials. Further inquiries can be directed to the corresponding author.

## Supplementary Materials

The following supporting information can be downloaded at: https://www.mdpi.com/article/10.3390/genes16080916/s1, Table S1: adducin_mutations.xlsx.

## Abbreviations

The following abbreviations are used in this manuscript:

ADD1	Adducin 1 gene
ADD2	Adducin 2 gene
ADD3	Adducin 3 gene
ADDA	α-adducin
ADDB	β-adducin
ADDG	γ-adducin
DARPP-32	Dopamine and cAMP-Regulated Phosphoprotein of 32 kDa
DynaMut2	Dynamic Mutation Analysis 2
mCSM	Mutation Cutoff Scanning Matrix
MutPred2	Mutation Prediction 2
PKA	Protein Kinase A
PKC	Protein Kinase C
Pmut	Pathogenic Mutation Predictor
PolyPhen-2	Polymorphism Phenotyping v2
Rb1	Retinoblastoma 1 Gene
Rho/ROCK	Rho-associated Protein Kinase Pathway
TAGAP	T-cell Activation GTPase-Activating Protein
